# The relationship between magnetic and electrophysiological responses to complex tactile stimuli

**DOI:** 10.1186/1471-2202-10-4

**Published:** 2009-01-15

**Authors:** Zhao Zhu, Johanna M Zumer, Marianne E Lowenthal, Jeff Padberg, Gregg H Recanzone, Leah A Krubitzer, Srikantan S Nagarajan, Elizabeth A Disbrow

**Affiliations:** 1 Biomagnetic Imaging Laboratory, Department of Radiology, University of California, San Francisco, San Francisco, CA 94143-0628, USA; 2 Center for Neuroscience, University of California, Davis, Davis, CA 95616, USA; 3 Section of Neurobiology, Physiology & Behavior, University of California, Davis, Davis, CA 95616, USA; 4 Department of Psychology, University of California, Davis, Davis, CA 95616, USA; 5 Department of Neurology, University of California, Davis, Davis, CA 95616, USA

## Abstract

**Background:**

Magnetoencephalography (MEG) has become an increasingly popular technique for non-invasively characterizing neuromagnetic field changes in the brain at a high temporal resolution. To examine the reliability of the MEG signal, we compared magnetic and electrophysiological responses to complex natural stimuli from the same animals. We examined changes in neuromagnetic fields, local field potentials (LFP) and multi-unit activity (MUA) in macaque monkey primary somatosensory cortex that were induced by varying the rate of mechanical stimulation. Stimuli were applied to the fingertips with three inter-stimulus intervals (ISIs): 0.33s, 1s and 2s.

**Results:**

Signal intensity was inversely related to the rate of stimulation, but to different degrees for each measurement method. The decrease in response at higher stimulation rates was significantly greater for MUA than LFP and MEG data, while no significant difference was observed between LFP and MEG recordings. Furthermore, response latency was the shortest for MUA and the longest for MEG data.

**Conclusion:**

The MEG signal is an accurate representation of electrophysiological responses to complex natural stimuli. Further, the intensity and latency of the MEG signal were better correlated with the LFP than MUA data suggesting that the MEG signal reflects primarily synaptic currents rather than spiking activity. These differences in latency could be attributed to differences in the extent of spatial summation and/or differential laminar sensitivity.

## Background

In the past two decades the use of noninvasive techniques to study the human brain has become pervasive. Data gathered from studies using positron emission tomography (PET) and functional magnetic resonance imaging (fMRI) have enhanced our knowledge of normal processing in the cerebral cortex, as well as deficits related to disease states. While these techniques are widely used to study aspects of brain organization and function, there are limitations regarding their use and the types of information that can be obtained. In particular, the poor temporal resolution of these two techniques limits studies primarily to the spatial domain.

A less widely used technique, magnetoencephalography (MEG), is a non-invasive method for detecting and characterizing changes in neuromagnetic fields in the brain. MEG has the advantage that it directly measures neuronal activity rather than a blood oxygenation based signal, an indirect measure of brain function that forms the basis of fMRI and PET measurements. MEG, therefore, has higher temporal resolution (on the order of milliseconds) with reasonable spatial resolution for cortical activity (on the order of millimeters) [1,2]. Since its introduction by Cohen in 1972 [3], MEG has proven useful for clinical applications and basic science research [1,2].

Despite its increasing popularity, the neurophysiological basis of the MEG signal is still not well established. Based on early *in vitro *electrophysiological studies in turtle cerebellar preparations [4-8], guinea pig hippocampal preparations [9-11] and a series of *in vivo *studies in rats [12-15], it has been hypothesized that the genesis of the signal is synchronous cellular currents emanating from parallel apical pyramidal dendrites [1,2]. Several computer modeling studies support this proposition [16-19]. It has also been proposed that weak, high-frequency magnetic responses observed in somatosensory cortex in humans [20,21] and piglets [22,23] may be related to action potential activity, though there is no direct electropysiological data to support this hypothesis. Further, there are limitations to the existing previous work.*In vitro *tissue preparations are limited because much of the cortical functional connectivity is lost and natural stimuli can not be used. In addition, previous animal studies using small numbers of gradiometer coils (1–4) to collect magnetic signals provide poor spatial resolution and make source localization challenging.

The bulk of existing data is based on studies using electrical stimuli [4-10,12-14,20-23] or penicillin-induced focal epilepsy [[12,13], and [15]]. These highly specialized stimulus conditions may not accurately reflect the relationship between MEG and the neural response under normal stimulus conditions. Further, the relationship between the MEG signal and the underlying neural response to complex natural stimuli has not been extensively investigated.

Our goal was to examine the relationship between the MEG signal and underlying neural activity in primary somatosensory cortex (S1 or 3b) in macaque monkeys using a complex natural stimulus consiting of varying rates of cutaneous stimulation. We used controlled mechanical stimuli administered at three different rates and directly compared the MEG response to two robust measures of neural activity, local field potentials (LFP), and multi-unit activity (MUA) in the same animals. We used varying rates of stimulation as a complex stimulus because the strength of the MEG and somatosensory evoked potential (SEP) response has been shown to decrease with increasing rate of tactile stimulation in human somatosensory cortex [24-35]. In addition, spiking activity in rat somatosensory cortex also shows a rate dependent effect [36-46]. We hypothesize that the MEG signal will faithfully reflect latency and amplitude characteristics of the underlying neural rate effect. Quantifying the reliability and validity of the MEG signal is crucial for interpreting existing MEG data, designing future experiments, and more fully appreciating cortical temporal processing.

## Results

In the present investigation, we examined the relationship between the MEG signal, local field potentials and multiunit activity in the same animal using calibrated mechanical stimuli delivered at varying rates of stimulation. As suggested in previous studies, the amplitude of the response was inversely related to the rate of stimulation for all three measures. The major findings in the present investigation were that 1) the relative amplitude of the MEG signal accurately reflected stimulus induced changes in underlying neural activity, however the amplitude of the MEG response was more closely correlated to the amplitude of the LFP than the MUA response; and 2) the response latencies of the LFP and MEG recordings were similar, while both were significantly different from the response latency of the MUA data.

### Magnetoencephalography

**Figure 1:**
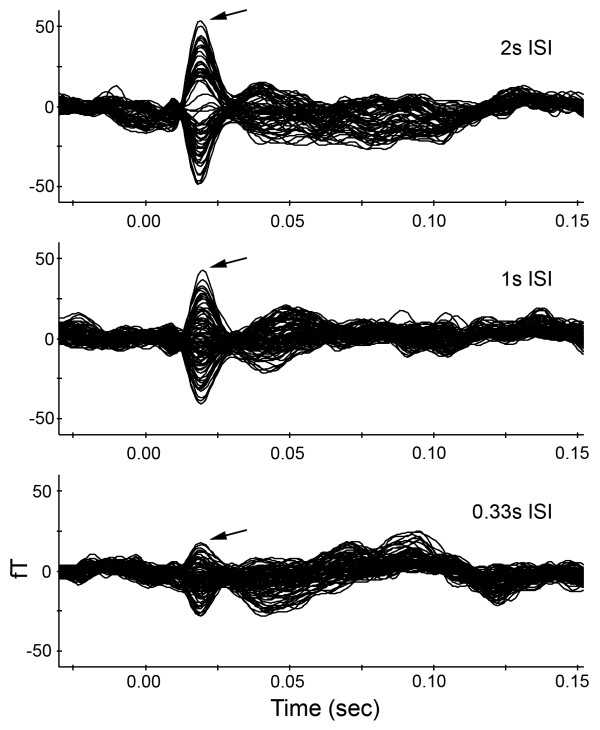
**An example of a somatosensory MEG response**. Simultaneous stimulation of right D1 and D2 evoked responses in the left hemisphere. The response waveforms at three stimulation rates are shown. From top to bottom, the interstimulus intervals (ISIs) were 2s, 1s and 0.33s. The filter range was 1–100 Hz. Each curve shows one channel's time course. The first response peak appeared at 19 ms (arrow). While the latency of this peak did not change across ISIs, the amplitude decreased with increasing stimulation rate.

A total of eleven sets of MEG data containing all three stimulation rates were collected from both hemispheres in each of two monkeys. Tactile stimuli simultaneously delivered to the index finger and thumb consistently evoked a significant change in the magnetic field relative to a prestimulus baseline at all three stimulation conditions in contralateral parietal cortex, but the amplitudes of the evoked responses were different at individual ISIs. Figure 1 shows an example recorded from one subject's left hemisphere.

**Figure 2:**
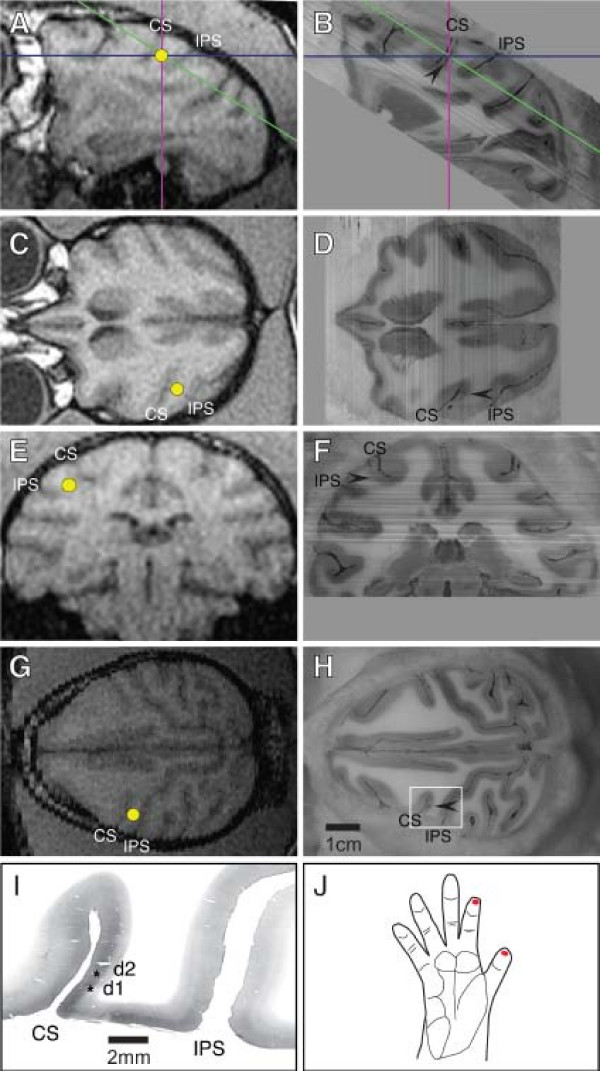
**An example of coregistration of MEG and electrophysiological signals**. Amira software was used to digitally reconstruct and "reslice" both MRI and histological sections, such that the locations of MEG signal sources and electrophysiological recording data could be identified with precision. A, C, E, Sagittal, horizontal, and coronal views, respectively, obtained using MRI in Case 24056. The yellow dot indicates the signal source identified using MEG. Blue, magenta, and green lines in panels A and B indicate planes of section shown in panels C-H. B, D, F: Sagittal, horizontal, and coronal views, respectively, that were digitally reconstructed from block face images obtained during histological sectioning of the brain. G: Horizontal view of the brain in Case 24056, digitally reconstructed from MRI data. Note that this plane of section matches the plane of section used for histological sectioning shown in panel H. H: Block face image of the brain taken during histological sectioning. The white box in panel H indicates the location of the photomicrograph shown in I. Arrowheads in panels B, D, F, and H indicate a single electrode track from recordings in the same location as the signal source identified using MEG. I: Digital photomicrograph of the section adjacent to the block face image shown in panel H, reacted for cytochrome oxidase. Asterisks indicate penetrations at which the receptive fields shown in panel J were obtained. The receptive fields at recordings in this location were identical for both MEG and electrophysiological recordings. The location of this penetration corresponds to the signal source identified using MEG. Digitally "resectioning" the MEG and histological data allowed us to match these data sets with high fidelity. Banding pattern in panels B, D, and F is due to lighting variations during photography of individual block face images.

The dominant waveform was a single large amplitude peak at about 20 msec. A similar large amplitude peak has been observed at around 40 ms in humans [for review, see [47]], which we assume corresponds to the large amplitude peak we measured in the monkey. Other peaks were also observed, but they were not consistently present. In four cases a small peak was seen prior to the large peak described above. A less consistent earlier small peak occurring around 20 ms has been evoked in humans using electrical stimulation [for review, see [47]] and using cutaneous stimulation [e.g. [48-50]]. This peak may correspond to the earliest small peak observed in the monkey data. The responses after the large peak were variable, and were not always present, which is also common in human data [51-55]. In this study, we focused on the consistent large peak, which is the most reliable and repeatable response. The dipole fit localized the source of this early peak response to the central sulcus (Figure 2A, C and 2E). All dipole sources were located within 1 cm of the electrophysiological recording sites in the digit representation of 3b.

**Figure 3:**
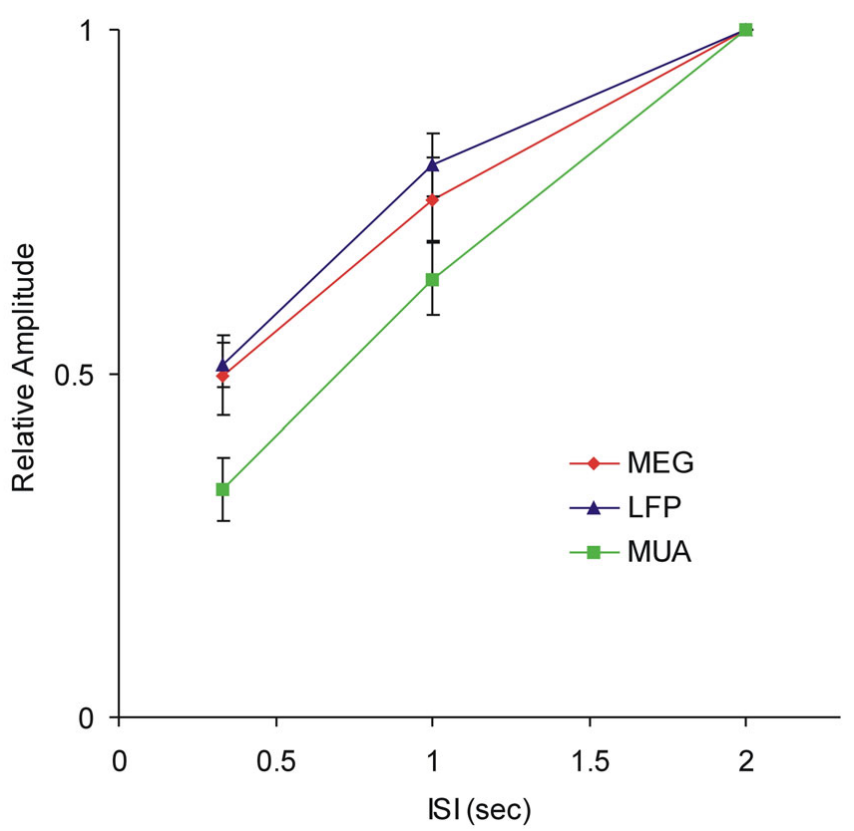
**Comparison of relative amplitude of MEG, LFP, and MUA data**. The relative response intensities for 1s ISI and 0.33s ISI were normalized to the 2s ISI signal amplitude. For all data sets, there is a significant change in the amplitude of response, regardless of the measure, with a change in the ISI of the stimulus, which constitutes different rates of stimulation. The relative amplitude of the MEG data was significantly different from the relative amplitude of MUA (p < 0.001), but not from the LFP (p > 0.05) relative amplitude.

The average response amplitude for all datasets from four hemispheres decreased with increasing stimulation rate as in the example shown in Figure 1. For the slow stimulation rate (2s ISI), the grand average response magnitude (RMS) was 24.2 ± 2.5 fT. The average magnitude of the response (RMS) was 18.6 ± 2.2 fT with an ISI of 1s and 13.4 ± 1.6 fT with the 0.33s ISI. The grand average moment response values (Q) were 11.5 ± 2.1, 8.7 ± 1.9, and 5.9 ± 1.4 (nAm) for 2s, 1s and 0.33s ISIs, respectively. For 1s and 0.33s ISIs, the grand average moment response values were 75.3 ± 5.9% and 49.7 ± 5.7% relative to the average moment response value at the 2s ISI. The amplitude of the response was significantly different for the three rates (p < 0.01). The relative intensity changes are shown in Figure 3.

**Figure 4:**
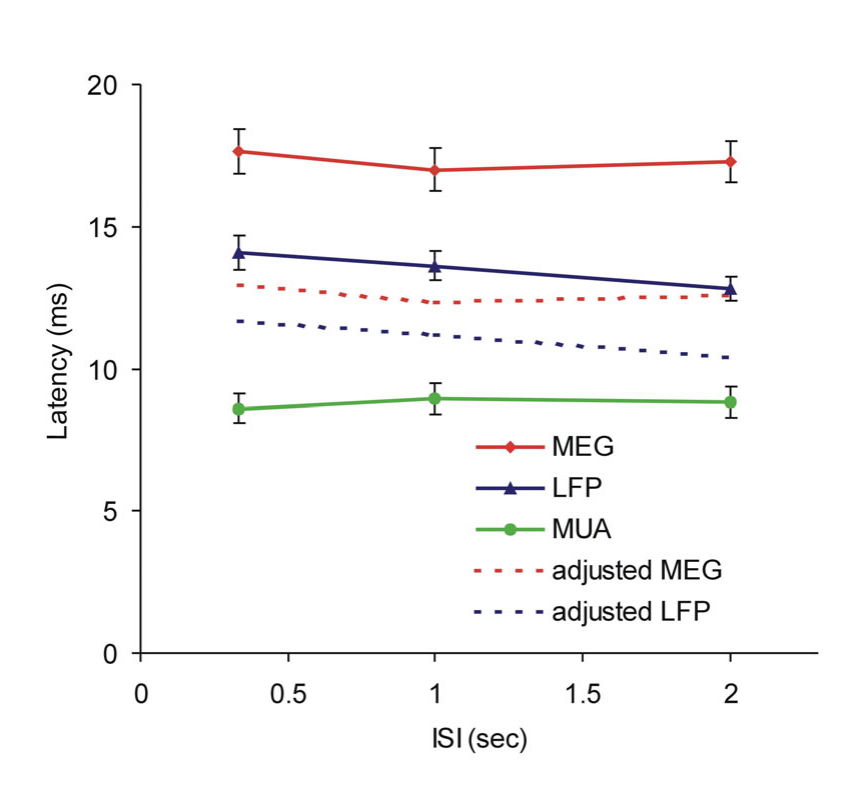
**Comparison of peak latency of MEG, LFP and MUA**. The absolute response peak latency with respect to stimulus onset was different for the three measures at each stimulation rate. Both original and adjusted latencies (the original peak latency minus the maximum time shift introduced by the cut-off frequency of the filter) were shorter in LFP than in MEG recordings. Both were longer than the peak latency of the MUA burst.

The average latency of the first large response peak for all datasets from the four hemispheres was 17.3 ± 0.7 ms, 17.0 ± 0.7 ms and 17.6 ± 0.8 ms at 2s, 1s and 0.33s ISI respectively. For all stimulation rates, the first peak occurred at a mean of 17.3 ± 0.8 ms. The response latencies were not significantly different for the three rates (p > 0.05; Figure 4). All dipoles of the early large response were located in the central sulcus of the hemisphere contralateral to the stimulated hand.

### Electrophysiological measures

**Figure 5:**
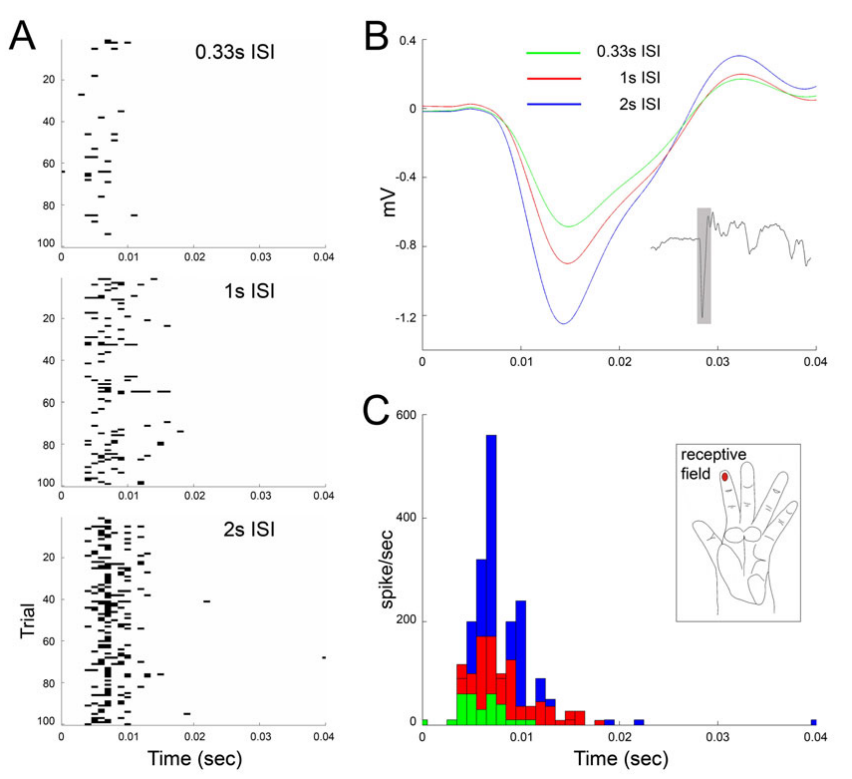
**An example of the rate effect in LFP and MUA recordings**. For MUA recordings, the same threshold was set for all trials recorded from the same cortical site. A spike was counted when the voltage was over the threshold level within a 1 ms bin. In A, raster plots of raw MUA data were collected over 100 trials from a single recording site, which is at the depth of 4000 μm in the central sulcus. Each dot represents a single spike. B shows the average LFP waveforms over 100 trials in the first 40 ms after the onset of the stimulus (as shown in the grey box in the inset LFP waveform at 2s ISI) at each stimulation rate recorded from this site. C shows the post-stimulus time histograms of the same data used in A. The green, red and blue lines in B and bars in C represent data at 0.33s, 1s and 2s ISIs respectively.

LFP and MUA data were recorded from the contralateral distal D1 and D2 representations in area 3b at twenty-eight sites from three hemispheres. The neurons recorded at these sites had clearly defined receptive fields on contralateral distal D1 or D2. Histological analysis indicated that all sites were within the caudal bank of the CS, approximately in layer 4 (Figure 2I). At each recording site, LFP and MUA data were measured simultaneously. A representative example from a single recording site is shown in Figure 5. LFP activity was averaged over 100 trials (Figure 5B) and MUA spikes were displayed as single trial rasters (Figure 5A) and PSTHs (Figure 5C).

The LFP waveform consisted of an initial, large, negative peak followed by a smaller positive peak. Similar waveforms have previously been recorded from layer 4 of monkey [56] and rat [42,57] somatosensory cortex. As with the MEG data, the amplitude of the LFP was also dependent on ISI, with the longest ISI resulting in the largest response amplitude (Figure 5B and 3). The average LFP magnitudes were 0.70 ± 0.1, 1.2 ± 0.2 and 1.5 ± 0.2 mV for 0.33s, 1s and 2s ISIs respectively. For 1s and 0.33s ISIs, the average magnitudes were 51.2 ± 3.1% and 80.4 ± 4.6% relative to the average magnitude at the 2s ISI.

In the example illustrated in Figure 5, little or no spontaneous activity was observed. Stimulus evoked MUA activity occurred with a latency of 6 ms post stimulus onset (from the peak in the PSTH). The duration of activity was brief in that there was an initial burst of activity 6 ms after stimulus onset, and then a rapid cessation of activity a few milliseconds later. As with the other metrics studied, there was a clear dependence of the magnitude of the response on ISI, with the greatest response at the 2s ISI and the weakest response for the 0.33s ISI (Figure 5A, 5C and 3). The average spike counts within 20 ms after the onset of the stimulus over the 100 trials were 98.3 ± 17.9, 199.4 ± 28.5 and 307.5 ± 36.3 for 0.33s, 1s and 2s ISIs respectively. For 1s and 0.33s ISIs, the average spike counts were 33.0 ± 4.6% and 63.7 ± 5.2% relative to the average magnitude at the 2s ISI.

### Comparison between MEG, LFP and MUA

The qualitative impressions from these data are that all three metrics show the highest activity at the longest ISI and the least activity at the shortest ISI. In order to quantify this assessment across the 28 recording sites, we computed the average MEG, LFP and MUA amplitudes. The early main peak amplitudes of the average LFP curves and the dipole moments (Q value) based on the early MEG response peaks were used for comparison. For the MUA data, the total spike count within 20 ms after the onset of the stimulus was used to evaluate the intensity of the MUA for all recording sites. In order to compare the three metrics (MEG, LFP and MUA), the response amplitudes at the 0.33s and 1s ISI were normalized to the response amplitude at the 2s ISI. These data are shown in Figure 3. The normalized amplitudes were significantly different across both ISI and measurement method (p < 0.001). Post-hoc tests indicated that MUA amplitude was significantly lower than LFP (p < 0.01) and MEG (p < 0.01) signal amplitude, while LFP and MEG results were not significantly different from each other (p > 0.05).

**Figure 6:**
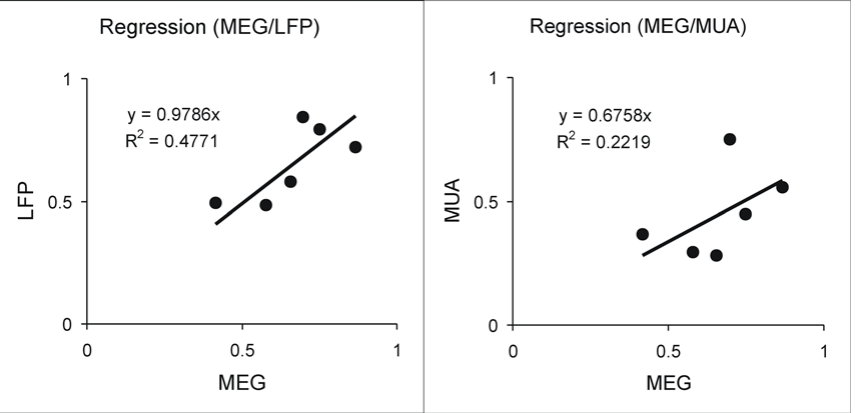
**Scatter plots for the regression analysis**. The response amplitudes at 0.33s and 1s ISIs are normalized to the response amplitude at the 2s ISI for each measurement method. Each plot shows the averaged amplitudes in one measurement method against another. The averaged amplitudes in one measurement metric recorded from three hemispheres are combined to make the scatter plots. The amplitudes at the 2s ISI are removed from the plots, since data were normalized to these values. A trend-line is fitted based on the six paired data points from the three hemispheres in each plot. The slope of the trendline and R^2 ^for the fit is shown in each plot.

The correlation between metrics was evaluated using regression analysis. The coefficient of determination (R^2^) from each hemisphere is listed in the table 1. The R^2 ^values show the greatest correlation between the MEG and LFP signal (0.79 to 1.00), while the MEG and MUA data are not as highly correlated (0.67 to 0.93). A scatter plot (Figure 6) using normalized data shows the pooled data from all hemispheres. The slope in the MEG/LFP comparison (0.98) is greater than the MEG/MUA comparison (0.68). These results indicate that MEG and LFP signals are more strongly correlated than the MEG and MUA signals.

**Table 1:** Coefficient of determination (R^2^) from regression analysis across metrics in each hemisphere.

	R^2^ hemisphere 1	R^2^ hemisphere 2	R^2^ Hemisphere 3
MEG/LFP	0.9979	0.785	0.9373
MEG/MUA	0.934	0.6676	0.8612

A pairwise regression analysis was used to evaluate the correlation between MEG and LFP/MUA. Regression parameters were computed between measurement methods in each hemisphere individually (for more details, see the text).

### Latency

Response peak latency did not significantly change with the rate of stimulation for MUA, LFP or MEG measures (p > 0.05). However, response latency was significantly different across measurement modalities (p < 0.01). The latency of the response was longest for the MEG signal, shorter for the LFP signal and shortest for the MUA signal (Figure 4).

As described in the Methods section, the cut off frequency of the filter could have introduced a phase delay. The maximum time delay for LFP and MEG data introduced by phase shifting was up to 2.4 ms and 4.7 ms respectively. Taking this delay into account, the mean adjusted latencies (original latency minus the maximum time delay) of the LFP and MEG peaks at each ISI were 11.1 ± 0.3 and 12.6 ± 0.4 ms respectively, while the mean peak response latency of the MUA data was 8.8 ± 0.5 ms (Figure 4, dashed lines). The mean adjusted latencies were significantly different across measurement modalities (p < 0.001), but not across ISIs. Post-hoc tests indicated that the adjusted latencies were significantly longer for MEG than for LFP data (p < 0.05), and both MEG and LFP latencies were significantly longer than the latency of the MUA burst (p < 0.001). The latency difference between the MEG and LFP data was much smaller than the latency difference between the MEG and MUA data. Therefore, as with measures of amplitude, the MEG data more closely reflected the LFP rather than the MUA response.

## Discussion

In this study, we used MEG to record magnetic field changes as a function of stimulation rate. For comparison we made LFP and MUA recordings from the same monkeys using identical stimuli. Our results demonstrate that the MEG signal faithfully reflects underlying neural activity. In addition, the MEG signal correlates more closely to LFP than MUA data in both amplitude and latency.

### Signal amplitude changes corresponding to varying stimulation rates

The relative response amplitude decreased in MEG, LFP and MUA signals with increasing stimulation rate, which is consistent with previous studies of human SEPs [24,27,29], human SEFs [25-29,32,33,35], rat SEPs [30,31] and rat electrophysiological recordings [36-46]. Because increasing stimulation rate reduced both amplitude of magnetic field and spike number in guinea pig hippocampal preparations, it has been suggested that the rate effect may be due to an increase of tonic inhibition at high stimulation rates [11].

The three methods used in our study showed a similar inverse relationship between stimulus rate and response magnitude, but to different degrees. MUA data showed the most pronounced rate effect, while LFP and MEG rate effect changes were not as pronounced. We also showed a stronger correlation between MEG and LFP than MEG and MUA signal amplitude changes across ISIs. Thus the MEG response more closely reflected the LFP rather than the MUA signal. Local field potentials are low-pass filtered electrophysiological signals that are generally considered to be the summed synaptic activity reflecting the slow component extracted from the neural response [e.g. [58-60]]. The fact that the relative amplitude decreased more in MUA than in LFP and MEG signals at low ISIs agrees with the linear property of post-synaptic activity and the nonlinearity of MUA which relies on the number of neurons involved and their threshold levels. These results directly demonstrate that MEG signal amplitude fluctuations are highly correlated with LFP rather than MUA amplitude fluctuations. Moreover, because of the similarities between the macaque monkey model and human cortex, our findings support the proposal that the human MEG signal reflects mainly post-synaptic activity [1,2], and MEG studies should be designed and interpreted in light of the fact that this signal does not appear to represent spike activity.

### Latency across measurement techniques

In the present investigation, the mean MUA peak burst latency was 8.8 ms, while the mean adjusted latency of the LFP and MEG signals were 11.1 ms and 12.6 ms, respectively. The LFP latencies observed in our study are consistent with those observed previously in layer 4 of rat [42,57] and monkey [56] somatosensory cortex. The first peak latency of electrical-stimuli-evoked SEPs recorded from the postcentral gyrus of anesthetized monkeys, which is around 10 ms, is slightly shorter than the adjusted latency of the first main LFP peak from our study of natural stimuli [56,61]. In contrast, the peak latency of the MUA bursts was 2.3 ms earlier than the adjusted peak latency of the LFP. The time delay of the LFP signal in our study was similar to that reported by Murthy and Fetz [62], who showed that electrical-stimuli-evoked multi-unit discharges occurred with the highest probability at about 2.7 ms earlier than the LFP peak in sensorimotor cortex of awake monkeys. A slightly longer (~5 ms) delay between MUA and LFP has also been reported in rat somatosensory cortex [42,63].

Previous studies have also demonstrated that the LFP signal not only has a slower onset, but can last up to tens of milliseconds compared to the action potential, which has a rapid onset and a short duration of about 1 millisecond [1]. There are several reasons why both LFP latency and length of response are longer than that of the MUA signal. First, both synaptic activity and other intrinsic dynamics such as membrane afterpotentials appear to contribute to the LFP [64], while only fast membrane potential change contributes to the MUA signal. Second, the extent of spatial summation of the LFP (0.5–3 mm) is much wider than that of the MUA signal (0.14–0.3 mm) [64]. MEG recordings presumably reflect summed signals from all local active neurons, which is likely much larger than the spatial extent of the LFP signal observed with a single electrode. This difference in spatial summation could account for the longer peak latency for the MEG vs. LFP and MUA data.

Further, the shorter peak latency of MUA may also be due to the cessation of neural activity caused by the poststimulus IPSPs from inhibitory interneurons, which underlie the in-field inhibition observed in monkey SEP studies [65]. For example, previous work shows that most spike activity terminated slightly before the early SEP component evoked using an airpuff stimulus [65]. The excitation of inhibitory interneurons may also contribute to population metrics like LFP and MEG signals, and make their peak latencies longer than MUA's.

It is also possible that the latency differences between MEG, LFP and spiking activity are due to differential laminar sensitivity of the measures. Generally the thalamic afferent signals arrive at the cortical granular layer first (layer 4), are relayed to supragranular layers, and then to infragranular layers [for review, see [66]]. Both EPSP latency [67] and BOLD MRI onset time [68] are shorter in layer 4 than in other layers. Action potentials recorded from rat somatosensory cortex showed that there was a delay of 3.5 ms and 4 ms from layer 4 to layer 3 and layer 5a/2, respectively [38]. A slightly shorter delay in spike latency from layer 4 to layer 2/3 (2 ms), and to layer 5a (3.5 ms) has also been reported in rats [69].

In the present study we recorded LFP and MUA data primarily in cortical layer 4, in which neurons have small receptive fields [70] and have robust responses [71], compared to neurons from other cortical layers. However, the stellate cells (the dominant neurons in layer 4) lack an apical dendrite [72], and the current dipoles originating from layer 4 stellate cells are much weaker than those from layer 2/3 and 5 pyramidal cells [18]. For these reasons the MEG signal is unlikely to originate from layer 4. It is known that MEG is primarily sensitive to the magnetic fields produced by currents tangential to the brain surface, and the synaptic currents along the apical dendrite trunks of large pyramidal cells located within the sulci are thought to be the primary source of the MEG signal [1,2], thus we can infer that the latency of our MEG signal is derived from pyramidal cells in layers 2/3 and 5 (in particular in layer 5 cells with large somas and long thick apical dendrites).

The slightly longer MEG latency may also be due to the spatial resolution limitations of MEG. As discussed above, MEG detects all currents tangential to the brain surface, In the central sulcus, somatosensory evoked signals primarily originate from area 3b [for review, see [47]], but may be generated in the adjacent areas 3a and 1. Therefore, the longer MEG latency may reflect the sum of signals from several adjacent areas with different latencies.

Finally, the stimulation rate-dependent amplitude decrease was greater for the MUA than LFP and MEG signals. This difference suggests that MUA, which measures the neural activity primarily in layer 4, is temporally coupled to stimulus rate, and that these neurons have a stronger response to rate change. On the other hand, LFP and MEG signals, which measure activity from other cortical layers, may reflect local cortical computations, and show a reduced response to change of stimulation rate. Taken together, the data suggest that the 3.8 ms delay of the adjusted MEG signal compared with the MUA signal roughly corresponds to the relay time from layer 4 to infragranular layers.

### Does MEG faithfully reflect neural responses to complex stimuli?

We have shown that MEG is an excellent tool for the *in vivo *study of neural activity in response to temporally complex stimuli. In particular, we observed a linear relationship between MEG and LFP data, with a decrease in response amplitude of approximately 80 and 50% for the 1s and 0.33s ISIs relative to the average magnitude at the 2s ISI. In contrast, the relationship between the blood oxygenation level dependent (BOLD) response and LFP and MUA data is more necessarily complex reflecting the interaction of local metabolism, blood oxygenation, volume, flow and intravascular magnetic susceptability. In fact, there is evidence that the relationship between the BOLD signal and various measures of neural activity at higher visual and tactile stimulation frequencies is not entirely linear [73-76].

We also examined latency differences across measurement techniques, but these discrepancies were relatively small (1.5 and 3.8 ms for MEG vs. corrected LFP and MUA data respectively). These differences are an order of magnitude smaller than the temporal resolution of the BOLD response, which is known to be around 4–8 seconds [77,78]. While efforts have been made to deconvolve the hemodynamic response [e.g. [79-81]], BOLD signals are significantly delayed relative to underly neural activity. In contrast, we have now shown that the MEG signal faithfully represents the temporal dynamics of underlying electrophysiological activity. Thus, MEG is a superior non-invasive imaging technique for studies involving stimuli that vary over short time scales.

## Conclusion

In the present study, the relationship between somatosensory evoked MEG, LFP and MUA signals recorded from the macaque monkey primary somatosensory cortex was investigated. We found that the amplitude of the response was inversely related to the stimulation rate, but to different degrees for the three techniques. The amplitude decrease at high rates of stimulation was significantly greater for MUA than LFP and MEG data. The latency of response was longest for the MEG signal and shortest for the MUA signal. Based on the correlation of intensity and latency between MEG and LFP signals, we conclude that the MEG signal reflects primarily synaptic currents rather than spiking activity. Because the MEG signal faithfully reflects complex stimulus properties, this non-invasive means of measurement is not only complementary to, but may also replace some invasive modalities, such as intracranial electrode recording. MEG can also help to bridge the gap between basic science research carried out in animals and human clinical and neuroscience findings.

## Methods

### Subjects

MEG, magnetic resonance imaging (MRI), and electrophysiological studies were performed in both hemispheres of two anesthetized adult male macaque monkeys (15 and 17 kg). All procedures in this study were approved by the UC Davis and UC San Francisco IACUCs, conformed to Society for Neuroscience Policy and followed the guidelines outlined in the Ethical Treatment of Animals (National Institutes of Health).

### Stimulation

Tactile stimulation was delivered through polyvinyl chloride tubing connected to a somatosensory generator (Somatosensory Stimulus System, 4-D Neuroimaging, San Diego, CA) which was outside of the recording room. Earplugs were used to block any sound generated by the stimulator. Pneumatically driven pulses (25 PSI) were applied simultaneously to the distal tip of digits 1 (thumb; D1) and 2 (index finger; D2) through a plastic spring clip with a balloon diaphragm (1 cm diameter). We delivered tactile stimuli to both D1 and D2 simultaneouosly to increase activated cortex and ensure a robust signal in the anesthetized preparation.

The duration of each stimulus was 140 ms with a rise and fall time of 30 ms. Three different interstimulus intervals (ISIs, time between the onset of stimuli) of 0.33s, 1s and 2s, were presented. These rates were chosen because neural responses have been shown to be rate dependent in this stimulus range. We refer to this rate varying stimulus as "complex" or "temporally complex" because the amplitude of response to an individual stimulus was influenced by the preceding stimulus. This stimulation protocol was used in both MEG and electrophysiological recording experiments. For the MEG experiments, each stimulus block included 512 individual stimuli at each rate. The block durations for 0.33s, 1s and 2s ISIs were 169s, 512s and 1024s, respectively and blocks were presented in random order with a 10 minute interval between stimulus blocks. For the electrophysiological recording experiments each stimulus block type included 100 individual stimuli. The block durations for 0.33s, 1s and 2s ISIs were 33s, 100s and 200s, respectively.

### MRI acquisition

At the beginning of each scan, anesthesia was induced with ketamine hydrochloride (10 mg/kg; IM), and maintained with the inhalation anesthetic Isoflurane delivered at 1–2% in O_2_. Atropine sulfate (0.04 mg/kg, IM) was administrated to reduce tracheal secretions, and lactated Ringer's solution (10 mL/kg/hr) was continuously infused (IV). Heart rate, respiration rate, and SpO_2 _were monitored throughout, and body temperature was maintained near 37°C. Once anesthetized, the animal's head was secured in an MR-compatible stereotaxic frame, and then the animal was placed in the MRI machine. Three fiducials were placed at landmark sites (the central forehead, left and right preauricular points) and were later used to co-register the MRI structural image with MEG data for magnetic signal source localization.

Each animal was scanned using a 1.5T MRI scanner (GE Medical System, Milwaukee, WI) with a 5" surface coil secured to the top of the skull perpendicular to the midline to acquire a 3D structural brain image (flip angle = 40°, TR = 27 ms, TE = 7 ms, FOV = 190 × 190 mm, 1.0 mm slice thickness, 256 × 256 × 124 pixels, in-plane resolution 0.74 mm × 0.74 mm).

### MEG acquisition and data processing

For MEG experiments, anesthesia was induced with ketamine hydrochloride. Anesthesia was maintained with a combination of ketamine hydrochloride (3–5 mg/kg) and midazolam (0.06 mg/kg) administered either intravenously or intramuscularly. Due to technical constraints we were not able to use Isoflurane in the MEG experiments, so the anesthesia for the electrophysiological and MEG experiments was not identical. The use of monitoring equipment within the shielded room caused interference with data collection, so the physiological parameters described above were monitored between runs, at 15 to 30 minute intervals. An experimenter stayed in the shielded room with the animal at all times to ensure that the animal did not move during data acquisition. MEG experiments were separately performed on each hemisphere of the two animals. In one animal data was acquired twice in one hemisphere and thrice in the other hemisphere. In the other animal data was acquired three times in each hemisphere. Individual experiments on the same animals were separated by at least two weeks.

MEG signals (somatosensory evoked fields or SEFs) were recorded using an Omega 2000 Whole-Cortex MEG System (CTF Systems Inc. Port Coquitlam, Canada; 275 DC SQUID first-order axial gradiometers). The MEG machine was positioned horizontally during the experiment. The anesthetized animal was placed on its side (contralateral to the site of stimulation). The animal's head position was manipulated such that the region over the contralateral central sulcus touched the posterior-superior wall of the sensor helmet. Three fiducials were placed at the same landmark sites used in the MRI scans for co-registration with structural MRI images and localization of the MEG signal source. To assess head movement, head position relative to the MEG sensors was determined before and after each test block. Data were collected at a sample rate of 1200 Hz. Five hundred and twelve trials were collected at each stimulation rate; only artifact-free trials were used for analysis. As previously reported in humans [51], we found that somatosensory evoked responses occurring later relative to stimulus onset (for example in secondary somatosensory cortex) were more anesthesia level dependent and more variable. Therefore, we only analyzed the large early peak response, which likely originates from area 3b [for review, see [47,82]].

Source localization was performed using parametric dipole fitting. A single dipole fit was used to determine the location of the MEG signal in the contralateral hemisphere as commonly used in human MEG data analysis (e.g. Figure 2A, C and 2E). Since the monkey's head was smaller than a human's head, only approximately 30 sensors were recording brain activity while the remaining sensors detected room noise and in some cases heartbeat artifact rather than brain signals. For each experiment (all ISIs collected on a given day) these 30 sensors matching the maximum (positive and negative) early responses on both sides of the signal source were chosen to determine the equivalent current dipole of the most dominant source at the 2s ISI. The position and orientation of the identified dipole for the 2s ISI were fixed. Then, this dipole was used to estimate the response amplitudes (dipole mement, Q) for all three ISI conditions. The response latencies and dipole moments (Q) were estimated based on the early response peaks for the different stimulation conditions. The grand average moment response values (Q) were compared at different ISIs by normalizing the 0.33s and 1s ISI conditions to the 2s ISI condition. This normalization facilitated comparison of the relative changes in MEG responses as a function of rate with the rate dependent changes observed in LFP and MUA data.

### Electrophysiological recording and data analysis

Following the MEG recordings, acute electrophysiological recording experiments were carried out. The anesthetic regime and monitoring were identical to those described for the MRI acquisition phase of these experiments. Once anesthetized, the animals were placed in a stereotaxic frame. An incision was made over the scalp and the skin was retracted. The temporal muscle was retracted, and a craniotomy was performed over the central sulcus. Dimethylpolysiloxane was placed over the exposed cortex to prevent desiccation. A digital photograph of the exposed cortex, including the central sulcus (CS), the post-central gyrus (PCG) and the intraparietal sulcus (IPS) was taken. This photograph was printed and used to mark electrode track locations relative to sulcal landmarks and blood vessels for later data analysis and reconstruction.

Tungsten microelectrodes designed to record from neural clusters (5 Mega Ohms; A-M Systems, Inc.) were lowered into the brain and neurons were recorded on the caudal bank of the CS, approximately in layer 4, and on the PCG (Figure 2D, H and 2I). First, the location of the hand representations in areas 3b, 1 and 2 were identified and then a rough topographic map was obtained. Receptive fields were determined by lightly stimulating the hand with fine probes, soft brushes, and deflection of hairs. At recording sites in which neurons were responsive to stimulation of the tips of contralateral D1 and D2, LFP (passband: 2–100 Hz) and MUA (passband: 0.3–10 kHz) were recorded at 500 μm intervals in the depth of the caudal bank of the CS. For these recordings, the stimuli were pneumatically driven pulses delivered to the tips of D1 and D2 simultaneously, as in the MEG experiments. To mark the electrode tracks at which LFP and MUA data were recorded the electrode was coated with a fluorescent tracer, Cascade Blue (Invitrogen, Carlsbad, CA), for later identification in histologically processed tissue.

Data was collected at 25 kHz. A TDT Data Acquisition Workstation with a RA4PA Medusa Preamplifier (Lowpass filter: 7.5 kHz, Maximum voltage in: +/- 4 mV) and the TDT BrainWare software (Tucker-Davis Technologies, Alachua, FL, US) were used for data collection. Custom-made code written in Matlab was used for data analysis. The multi-units were identified based on responses heard on an audio monitor as well as visual confirmation on a real time display. Spikes were selected after manually adjusting the threshold offline to primarily include stimulus driven activity. The same threshold was used for spike acquisition from a single recording site for all three stimulation rates. A spike was counted when the voltage was over the threshold level within a 1 ms-bin. All of the spikes in the 100 trials (using 1 ms bins) could then be displayed as single trial rasters (e.g. Figure 5A) and post-stimulus time histograms (PSTH; e.g. Figure 5C).

The LFP or MUA data for each ISI from the 100-trials collected at each recording site were averaged. The early main peak amplitudes and latencies of the average LFP curves were used for comparison. At all 28 recording sites, neurons fired with a burst pattern starting with a short latency and stopping within 20 ms after the onset of the stimulus. The total spike count within 20 ms after the onset of the stimulus over the 100 trials and the burst peak (the peak in the PSTH) latency were compared with other measures.

ANOVA was used to assess statistical significances between responses across different stimulation conditions and measurement modalities. Post-hoc Tukey tests were performed to assess significance between specific condition pairs. Data are presented as mean values ± standard error of the mean throughout.

To evaluate the correlation between MEG and the electrophysiological measures we did a pairwise regression analysis comparing averaged MEG with MUA and LFP data from each hemisphere. For each measurement method, three averaged data points corresponding to 0.33, 1 and 2s ISIs from each hemisphere were used for paired comparisons. Regression parameters were computed between measurement methods in each hemisphere individually. The coefficient of determination (R^2^) was used to compare the correlation between each measure. To enable direct comparison of the measures, the response amplitudes at the 0.33s and 1s ISI were normalized to the response amplitude at the 2s ISI. Normalized amplitudes from all three hemispheres were combined in a scatter plot and were fitted with a trend-line. The slopes of the trend-lines and R^2 ^values in the two plots were compared to evaluate the correlation between different metrics.

### Filter simulation

The digital filters used in processing the MUA, LFP and MEG data may have introduced a phase delay. To examine this possibility, we simulated the filterbanks used in the experiments in MATLAB. Three filterbanks corresponding to MUA, LFP and MEG data collection parameters were created to replicate those used in data collection. Due to a potential confound in identifying the phase shift in the raw data, which has a complex frequency spectrum, test signals were used for simulations. Test signals were chosen in the passband of the filterbanks for each measure. These simulations were repeated for a number of test signals in the pass band of each filterbank. The filtered signal was compared with the test signal and the phase delay was calculated. The maximum time delays (based on peak latency, for the best ratio of signal to noise) introduced by the phase shifting properties of the filters used in collecting LFP and MEG data were 2.4 and 4.7 ms, respectively. There was no phase delay found in the MUA data.

### Histological processing

At the end of the electrophysiological recording experiment, three reference probes made from the plastic portion of a 20 gage IV catheter were placed into the brain for later alignment of histological and electrophysiological data sets. The animals were then euthanized and perfused transcardially with 0.9% saline, followed by 4% paraformaldehyde in phosphate buffer, and then 4% paraformaldehyde in 10% sucrose phosphate buffer. The brains were immersed overnight in 30% sucrose in phosphate buffer and then cut into 80-μm axial sections, using a freezing microtome. Block face images were acquired for every section with a Nikon CoolPix 5700 digital camera. Alternating sections were processed for cytochrome oxidase, Nissl substance, myelin, or fluorescent microscopy.

### Co-registration of MEG, MRI and electrophysiology data sets

Three fiducials were placed at the same three landmarks for both MRI and MEG scans. The locations of these fiducials (the central forehead, left and right preauricular points) were used to compute an affine transformation between head coordinates and the MR image. MEG sensor coordinates and orientations were then transformed to MRI coordinates using this affine transformation.

Electrophysiological recordings were related to cortical architectonic boundaries by aligning the probe location marked on the digital image during electrophysiological recording experiments with these same probes identified in histologically processed tissue. We aligned histological and electrophysiological data sets and confirmed that our recordings were in area 3b. To directly relate the location of these electrophysiological recordings in 3b to the MEG data, recording sites marked on the digital image of the brain were transposed onto an image of the dorsolateral view of the brain by aligning sulci and probes. A high resolution MRI image of a dorsolateral view of the brain with the source of the MEG response was coregistered with a digital image of the same view of the physical brain containing the electrophysioloical recording sites using sulcal landmarks. The region of interest was located between several distinct sulci including the central sulcus, the intraparietal sulcus, the post central dimple and the lateral sulcus. The MRI data and the entire series of block face images acquired during sectioning were manually coregistered using the Amira software package (Mercury Computer Systems, Chelmsford MA).

Using Amira, the block face images were manually aligned to correct for minor mismatches due to vibrations of the camera during sectioning. The resulting brain volume was then digitally "resectioned" in order to obtain a precise match of planar orientations between the MRI brain volume and the histological brain volume. Electrode tracks from electrophysiological recordings were identified in the same region in the central sulcus as the MEG signal source (e.g., Figure 2). The co-registration of MEG and MRI indicated that the MEG signal source was localized in the central sulcus and correlated well with the electrophysiological recording sites in area 3b.

## Abbreviations

MEG: magnetoencephalography; LFP: local field potentials; MUA: multi-unit activity; ISIs: inter-stimulus intervals; MRI: magnetic resonance imaging; PET: positron emission tomography; SEPs: somatosensory evoked potentials; SEFs: somatosensory evoked fields; RMS: root mean square; PSTH: post-stimulus time histogram; BOLD: blood oxygenation level-dependent; S1: primary somatosensory cortex; D1: thumb; D2: index finger; CS: central sulcus; PCG: post-central gyrus; IPS: intraparietal sulcus.

## Authors' contributions

ZZ, SSN and EAD designed the study. ZZ and EAD drafted the manuscript. JMZ, GHR, LAK and SSN revised the manuscript. ZZ, JMZ, MEL and EAD recorded and analyzed MEG and MRI data. ZZ, JMZ MEL, JP, GHR, LAK and EAD recorded LFP and MUA data. ZZ and JMZ analyzed LFP and MUA data. JP, MEL, ZZ and EAD performed the histological processing, JP and LAK coregistered histological and electrophysiological data sets. All authors read and approved the final manuscript.
